# Association of Neutrophil‐to‐Lymphocyte Ratio With All‐Cause and Cardiovascular Mortality Among Individuals With Depression: A Large‐Scale Cohort Study

**DOI:** 10.1002/brb3.70983

**Published:** 2025-10-15

**Authors:** Yue Chai, Guoxin Wang, Shumin Zhu, Congzhen Wei, Runsen Du, Zining Liu, Shuo Zhao, Li Yang, Yulan Geng

**Affiliations:** ^1^ Department of Laboratory Medicine The First Hospital of Hebei Medical University Shijiazhuang Hebei China; ^2^ Hebei Innovation Center of Clinical Medical Laboratory Technology Shijiazhuang Hebei China; ^3^ General Surgery The First Hospital of Hebei Medical University Shijiazhuang Hebei China; ^4^ Central Laboratory The First Hospital of Hebei Medical University Shijiazhuang Hebei China

**Keywords:** all‐cause mortality, cardiovascular diseases mortality, depression, National Health and Nutrition Examination Survey (NHANES), neutrophil‐lymphocyte ratio (NLR)

## Abstract

**Background:**

The relationship between mortality in depressed patients and the neutrophil‐to‐lymphocyte ratio (NLR) is not well‐documented.

**Methods:**

This cohort study, involving 8749 individuals diagnosed with depression, was derived from the National Health and Nutrition Examination Survey (NHANES) 2005–2018. Data on mortality were obtained by linking the cohort database to the National Death Index, with updates available as of December 31, 2019. Various analytical techniques, including Cox proportional hazards models, restricted cubic splines, Kaplan‐Meier curves, and subgroup and sensitivity analyses, were employed.

**Results:**

During an average follow‐up period of 84 months, 1023 participants (11.7%) died, with 271 of these deaths attributed to cardiovascular diseases (CVD). Our analysis demonstrated a positive connection among the NLR as well as mortality risk in all participants. In contrast to participants in the low NLR category (NLR ≤ 3.06), individuals belonging to the high NLR category (NLR > 3.06) exhibited a 64% increased risk of all‐cause mortality (hazard ratio [HR] 1.63, 95% confidence interval [CI] 1.36–1.95) and a 144% elevated risk of mortality due to CVD (HR 2.44, 95%CI 1.77–3.36) after multivariate adjustment. The interactions and data stratification supported the credibility of our results. Importantly, we discovered notable interactions within subgroups that were differentiated by age and diabetes.

**Conclusions:**

Elevated levels of the NLR are linked to a heightened risk of mortality from all causes, including CVD, among adults suffering from depression. This correlation is particularly pronounced in younger populations and in individuals who have diabetes.

## Graphical abstract

In this cohort study, an elevated neutrophil‐to‐lymphocyte ratio (NLR) was significantly associated with an increased risk of all‐cause and cardiovascular mortality among adults with depression. The correlation was particularly pronounced in younger individuals and those with diabetes. Our findings suggest NLR may serve as a valuable prognostic marker in this high‐risk population.

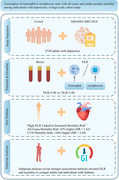

**Abbreviations**
95% CI: 95% confidence interval
BMI: body mass index
CVD: cardiovascular disease.
eGFR: estimated glomerular filtration rate
HDL: high‐density lipoprotein cholesterol
HR: hazard ratio
NHANES: National Health and Nutrition Examination Survey
NLR: neutrophil‐to‐lymphocyte ratio
PIR: the ratio of family income to poverty
TC: total cholesterol

## 1 Introduction

Depression, which ranks among the most common mental health disorders worldwide, affects approximately 300 million individuals (Marwaha et al. 2023; World Health Organization 2017). It is significantly linked to a reduction in life expectancy and an elevated risk of mortality (Chesney et al. 2014). In recent years, several scientific and clinical investigations have focused on investigating the intricate pathophysiology of this condition (Belzung et al. 2015; Chang et al. 2022; Spellman and Liston 2020). Among them, abnormalities in immune regulation (especially inflammatory response) have been shown to possibly play a central role in its pathophysiological processes (Beurel et al. 2020). In view of this, the identification of controllable risk factors is important for the prevention of depression‐related adverse outcomes and premature death.

Neutrophil‐to‐lymphocyte ratio (NLR), being a highly efficient and cost‐effective indicator of systemic inflammation, is more suitable for clinical practice than complex scoring systems (Zahorec 2021). In recent years, the NLR has gained attention for its predictive value in a variety of chronic inflammatory diseases and mood disorders. This indicator significantly influences the initiation, advancement, and prognosis of illnesses. Notably, the association between neuroinflammatory mechanisms and mental disorders has become a hot research topic. Numerous investigations have shown a substantial correlation between high NLR levels and mental illnesses, particularly depression. However, previous studies have limitations such as insufficient sample size, inadequate correction for key confounding variables, and lack of comprehensive mortality analysis for depressed populations.

Although the correlation between NLR and depression has been widely validated, its impact on patients’ long‐term prognosis is unclear. Based on it, this study utilized a representative sample of depressed patients from the NHANES 2005 up to 2018 for exploring the link between NLR and the risk of all‐cause and CVD mortality through a cohort design with an aim of providing an evidence‐based basis for clinical decision‐making.

## 2 Methods

### 2.1 Study Population

Data obtained from the NHANES, comprising a series of cohort surveys that reflect the national demographics, were utilized, with all participants providing written informed consent (Bhargava et al. 2025). The Centers for Disease Control and Prevention (CDC) has managed this data, which can be accessed publicly via the National Center for Health Statistics (NCHS). In accordance with the regulations established by CDC‐NCHS, this research was exempt from an ethical review. Two main components make up the project: a survey of home interviews and a mobile examination center (MEC) standardized physical examination (Johnson et al. 2013).

**FIGURE 1:**
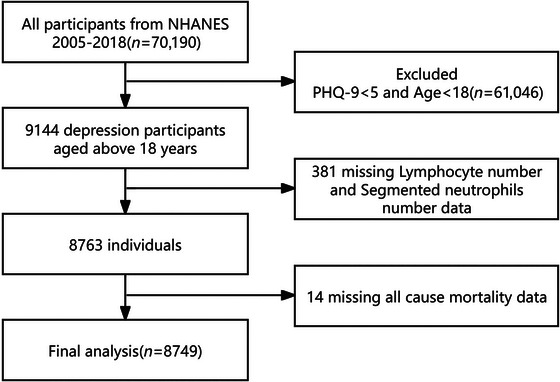
Flow chart of participant selection.

Based on the NHANES data from 2005 up to 2018, we constructed a cohort. The research originally included 70,190 participants who had an interview and MEC assessment. According to predetermined criteria, we removed participants who (1) were under 18 years of age; (2) had less than 5 Patient Health Questionnaire‐9 (PHQ‐9) scores (*n = * 61,046); (3) had incomplete lymphocyte or neutrophil count data (*n* = 381); and (4) had absent information on all‐cause mortality (*n = *14). The final research cohort included 8749 eligible participants, corrected for NHANES sample weights, representing 47,571,152 individuals in the U.S. adult population. The flowchart for screening research participants is shown in Figure 1.

### 2.2 Calculation of NLR

Using the complete blood count (CBC) measured through automatic hematological analyzer (Beckman Coulter, Miami, US), NLRs were calculated. The calculation formula was neutrophils count/lymphocytes count (X. Zhang et al. 2024).

### 2.3 Ascertainment of Depression

This research evaluated depressive conditions using the PHQ‐9, which the participants filled out during the interview phase of the MEC section of the NHANES program. As a widely recognized tool for assessment, the PHQ‐9 corresponds with the criteria outlined in the Diagnostic and Statistical Manual of Mental Disorders (DSM)‐IV for identifying depression (Kroenke et al. 2001). Each of the nine questions is evaluated on a scale that ranges from 0 to 3, capturing patient feedback: “not at all,” “a few days,” “more than half the days,” and “almost every day.” The cumulative score, varying between 0 and 27, is utilized to assess the depressive symptoms reported by individuals over the last 2 weeks (Kroenke and Spitzer 2002). Based on the classifications set forth by PHQ‐9, we categorized scores between 0 and 4 as belonging to the asymptomatic group, whereas scores from 5 to 27 were classified as symptomatic (Y. Wang et al. 2024).

### 2.4 Ascertainment of Outcomes

In this study, we obtained information on mortality status and causes by matching data from NHANES participants with the National Death Index (NDI) records up to the end of 2019. This data was obtained from the CDC website. The NDI system uses a nine‐category classification to record causes of death, of which we focused on two primary outcome indicators: (1) all‐cause mortality, encompassing all deaths regardless of their underlying causes, and (2) mortality due to CVD, which is explicitly defined as fatalities stemming from heart disease (UCOD_LEADING = 001) or cerebrovascular disease (UCOD_LEADING = 005) (S. Liu et al. 2024).

### 2.5 Assessment of Covariates

This analysis synthesized many potentially confounding factors based on established clinical recommendations and pertinent literature data(Mao et al. 2024; W. Wang et al. 2024; J. Yin et al. 2023; Y. Yin et al. 2025; X. Zhang et al. 2024). The research team employed a standardized household interview questionnaire to gather extensive sociodemographic data, including age (continuous variable), gender distribution (male/female), race and ethnicity (non‐Hispanic white, non‐Hispanic Black, Mexican American, and other race), educational attainment (less than high school, high school graduation, college and above), marital status (married/cohabiting or single), household economic status (evaluated via poverty income ratio [PIR]), as well as smoking status and self‐assessed health status. At the mobile health screening center, professionals standardized the measurement of fundamental physical parameters, including height and weight, for each study participant, from which the body mass index (BMI = weight [kg]/height^2^ [m^2^]) was calculated. In this study, BMI was used to categorize weight status into three classifications: healthy weight (<25 kg/m^2^), overweight (25–30 kg/m^2^), and obesity (≥ 30 kg/m^2^). Income was categorized into three categories based on the family PIR: low‐income group (≤ 1.3), medium‐income group (1.3–3.5), and high‐income group (> 3.5) (Ruan et al. 2022).

Smoking behavior was classified into three categories: non‐smokers (lifetime smoking < 100 cigarettes), past smokers (cumulative smoking ≥ 100 cigarettes but have ceased), and current smokers (cumulative smoking ≥ 100 cigarettes and are presently smoking). Alcohol use was evaluated by two separate 24‐h food recall interviews, and those who reported alcohol use in each interview were classified as alcohol consumers. Laboratory assessments included glycosylated hemoglobin (HbA1c), high‐density lipoprotein cholesterol (HDL‐C), and total cholesterol (TC) levels, while renal function was evaluated using the CKD‐EPI algorithm to determine eGFR (Inker et al. 2021). The diagnosis of diabetes mellitus was established using any of the following criteria: a clinically established diagnosis, HbA1c levels of 6.5% or higher, fasting glucose levels of 7.0 mmol/L or greater, random glucose levels of 11.1 mmol/L or above, or the use of medication aimed at lowering glucose (Tang et al. 2024). Furthermore, the diagnoses of cardiovascular diseases (such as coronary artery disease, heart failure, myocardial infarction, and stroke) and cancer were verified using medical records. The identification of hypertension relied on meeting at least one of these criteria: (1) systolic blood pressure of 140 mmHg or higher and/or diastolic blood pressure of 90 mmHg or higher, confirmed through three separate measurements; (2) a history of hypertension verified by a healthcare professional; or (3) current use of antihypertensive medication (Gu et al. 2023). The use of antidepressants was categorized as “no” for participants who were not using any medications or were on medications that were not antidepressants, and as “yes” for those who were using antidepressants (Table S1).

### 2.6 Statistical Analysis

This research used a statistically rigorous analysis using the NHANES‐recommended complex sampling design, including weighted adjustments, stratified sampling, and cluster analysis to guarantee that the findings accurately reflect the characteristics of the U.S. national population (Liao et al. 2024). Following the official NHANES guidelines, we utilized weighted data to delineate the fundamental characteristics of the study population. Continuous variables were represented as weighted mean ± standard error (SE), whereas categorical variables exhibited the raw number of cases and weighted percentages. For group comparisons, the χ^2^ test was used for categorical data, while the ANOVA or K‐W test was used for continuous data, contingent upon the distribution features. Using the maximum choice rank statistics approach from the R software maxstat package (Dong et al. 2023; Seckinger et al. 2012; L. Zhang et al. 2023), we established the appropriate cutoff value of NLR for forecasting survival outcomes and then classified the research population into high and low NLR groups. To assess the robustness of the NLR cutoff identified by the maxstat package, we performed a sensitivity analysis by categorizing NLR into quartiles (Q1: ≤ 1.5, Q2: 1.5–2.0, Q3: 2.0–2.7, Q4: > 2.7) and testing for trend in both all‐cause and cardiovascular mortality models using Cox proportional hazards regression, with Q1 as the reference group. Multiple imputation was performed for all variables with missing data using the Multiple Imputation by Chained Equations (MICE) method with 5 imputations. This approach was chosen to preserve statistical power and minimize potential selection bias.

This research used the RCS model to draw HR curves in order to examine the potential nonlinear dose‐response connection between NLR and mortality risk. To elucidate the independent link between NLR and both all‐cause as well as CVD deaths in patients with depression, we developed a weighted Cox proportional hazards regression model with four sequential adjustments: model 1 (unadjusted); model 2 (adjusted for demographic variables: age, gender, ethnicity, education level, marital status, and family income according to PIR); model 3 (further adjusted for BMI, HbA1c, HDL‐C, TC, eGFR, smoking status, and alcohol drinking status); and model 4 (fully adjusted model : additionally adjusted for diabetes, history of cardiovascular disease, cancer, and PHQ‐9 score as a continuous variable). Multicollinearity among covariates was assessed using variance inflation factors (VIFs). A VIF ≥ 5 was considered indicative of substantial multicollinearity. The proportional hazards assumption was evaluated using Schoenfeld residuals for each covariate in both all‐cause and cardiovascular mortality models. A *p* value > 0.05 was considered to indicate no violation of the assumption. Covariates were selected a priori based on established clinical knowledge and consistent evidence from prior epidemiological studies linking these factors to systemic inflammation, immune function, and mortality risk (Chu et al. 2025; H. Liu et al. 2025; Mahemuti et al. 2023; Sun et al. 2025; Yao et al. 2024; X. Zhang et al. 2024). To assess the independence of the association between NLR and mortality, a hierarchical adjustment strategy was employed. This approach sequentially accounts for demographic, lifestyle, clinical, and disease‐specific factors, allowing for a transparent evaluation of how the NLR–mortality association evolves with progressive control of potential confounders.

The Kaplan‐Meier technique was used to evaluate survival probabilities in patients with varying NLR levels, and group comparisons were conducted using log‐rank testing. Subgroup analyses were categorized by age (<65 years/≥65 years), gender, race, smoking status, diabetes, hypertension, and cancer, with interactions being assessed. All analyses were performed utilizing R software (version 4.2.1) alongside Free Statistics software (version 2.1.1) (Y. Wang et al. 2023; Y. Yin et al. 2025), with statistical significance established at a two‐sided α = 0.05.

## 3 Results

### 3.1 Baseline Characteristics

This research included 8749 individuals with depressive disorders (mean age 46.34 years; 40.0% male). Survival analysis determined that the optimal cutoff value for NLR was 3.06 (Figure S1, Supporting Information). Based on this criterion, participants were categorized into a high NLR group (>3.06, *n = *1485) and a low NLR group ≤ 3.06, *n* = 7264). Comparative analysis demonstrated that the elevated NLR group had distinct clinical and demographic characteristics, including older age, a higher proportion of White individuals, and increased prevalence of diabetes mellitus, cancer, and CVD. Moreover, this group exhibited a greater proportion of smokers, along with significantly reduced total cholesterol levels, lymphocyte counts, and eGFR (Table 1).

**TABLE 1:** Baseline characteristics of the study participants in the NHANES 2005–2018 cycles^a^.

Characteristic	Total (*n* = 8749)	Lower NLR (*n* = 7264)	Higher NLR (*n* = 1485)	*p*‐value
Age, mean (SE), years	46.34 (0.30)	45.43 (0.31)	50.75 (0.58)	< 0.001
Gender, *n* (%)				0.31
Male	3520 (40.00)	2850 (40.00)	670 (42.00)	
Female	5229 (60.00)	4414 (60.00)	815 (58.00)	
Race/Ethnicity, *n* (%)				< 0.001
Non‐Hispanic White	3703 (65.69)	2892 (63.88)	811 (74.55)	
Non‐Hispanic Black	1876 (12.11)	1676 (13.20)	200 (6.76)	
Mexican American	1436 (8.77)	1234 (9.21)	202 (6.59)	
Other Race	1734 (13.43)	1462 (13.71)	272 (12.10)	
Education level, *n* (%)				0.52
Less than high school	1065 (6.57)	894 (6.67)	171 (6.06)	
High school or equivalent	3715 (40.50)	3050 (40.23)	665 (41.79)	
Greater than high school	3959 (52.93)	3314 (53.09)	645 (52.16)	
Marital status, *n* (%)				>0.99
married or living with a partner	4212 (54.08)	3464 (54.08)	748 (54.10)	
living alone	4141 (45.92)	3444 (45.92)	697 (45.90)	
PIR, *n* (%)				0.50
low income	3498 (32.32)	2913 (32.60)	585 (30.96)	
medium income	2960 (38.53)	2448 (38.58)	512 (38.29)	
high income	1542 (29.15)	1277 (28.82)	265 (30.76)	
BMI, mean (SE), kg/m^2^	30.12 (0.14)	29.95 (0.16)	30.94 (0.27)	0.046
BMI, *n* (%)				0.47
< 25	2308 (27.35)	1938 (27.58)	370 (26.23)	
25–30	2455 (28.72)	2047 (28.93)	408 (27.67)	
>30	3876 (43.93)	3207 (43.49)	669 (46.10)	
HbA1c, mean (SE), %	5.69 (0.02)	5.68 (0.02)	5.76 (0.03)	0.15
HDL, mean (SE), mmol/L	1.36 (0.01)	1.36 (0.01)	1.38 (0.02)	0.20
TC, mean (SE), mmol/L	5.04 (0.02)	5.07 (0.02)	4.92 (0.04)	0.001
Neutrophil, mean (SE), ×10^9^/L	4.56 (0.03)	4.18 (0.03)	6.39 (0.07)	< 0.001
Lymphocyte, mean (SE), ×10^9^/L	2.24 (0.03)	2.37 (0.03)	1.61 (0.02)	< 0.001
eGFR, mean (SE), mL/min/1.73m^2^	96.55 (0.41)	97.40 (0.40)	92.36 (0.80)	< 0.001
Smoking status, *n* (%)				< 0.001
Never	3993 (45.30)	3386 (46.40)	607 (39.97)	
Former	1969 (24.18)	1541 (23.21)	428 (28.90)	
Current	2475 (30.52)	2063 (30.39)	412 (31.13)	
Drinking status, *n* (%)				0.19
No	5510 (68.37)	4552 (67.95)	958 (70.45)	
Yes	2099 (31.63)	1769 (32.05)	330 (29.55)	
Hypertension, *n* (%)	3981 (41.92)	3166 (39.48)	815 (53.81)	< 0.001
Diabetes, *n* (%)	1972 (17.57)	1534 (16.13)	438 (24.59)	< 0.001
Cancer, *n* (%)	844 (10.42)	624 (9.60)	220 (14.42)	< 0.001
History of CVD, *n* (%)				< 0.001
No	7531 (88.46)	6380 (89.86)	1151 (81.62)	
Yes	1218 (11.54)	884 (10.14)	334 (18.38)	
Antidepressant use, *n* (%)				< 0.001
No	7053 (77.35)	5939 (78.40)	1114 (72.21)	
Yes	1696 (22.65)	1325 (21.60)	371 (27.79)	

^a^ Categorical variables are expressed as unweighted number (weighted percentage), while continuous variables are reported as mean (standard error). Among the 8749 patients, the amount of missing values for the covariates were 110 (1.26%) for BMI, 1140 (13.03%) for drinking status, 10 (0.11%) for education level, 169 (1.93%) for eGFR, 28 (0.23%) for HbA1c, 134 (1.53%) for HDL, 396 (4.53%) for marital status, 749 (8.56%) for PIR, 312 (3.57%) for smoking status, 170 (1.94%) for TC.

Abbreviations: BMI, body mass index; CVD, cardiovascular disease; eGFR, estimated glomerular filtration rate; HDL, high‐density lipoprotein cholesterol; NHANES, National Health and Nutrition Examination Survey; NLR, neutrophil‐to‐lymphocyte ratio; PIR, the ratio of family income to poverty; TC, total cholesterol.

### 3.2 NLR and All‐Cause Mortality

**FIGURE 2:**
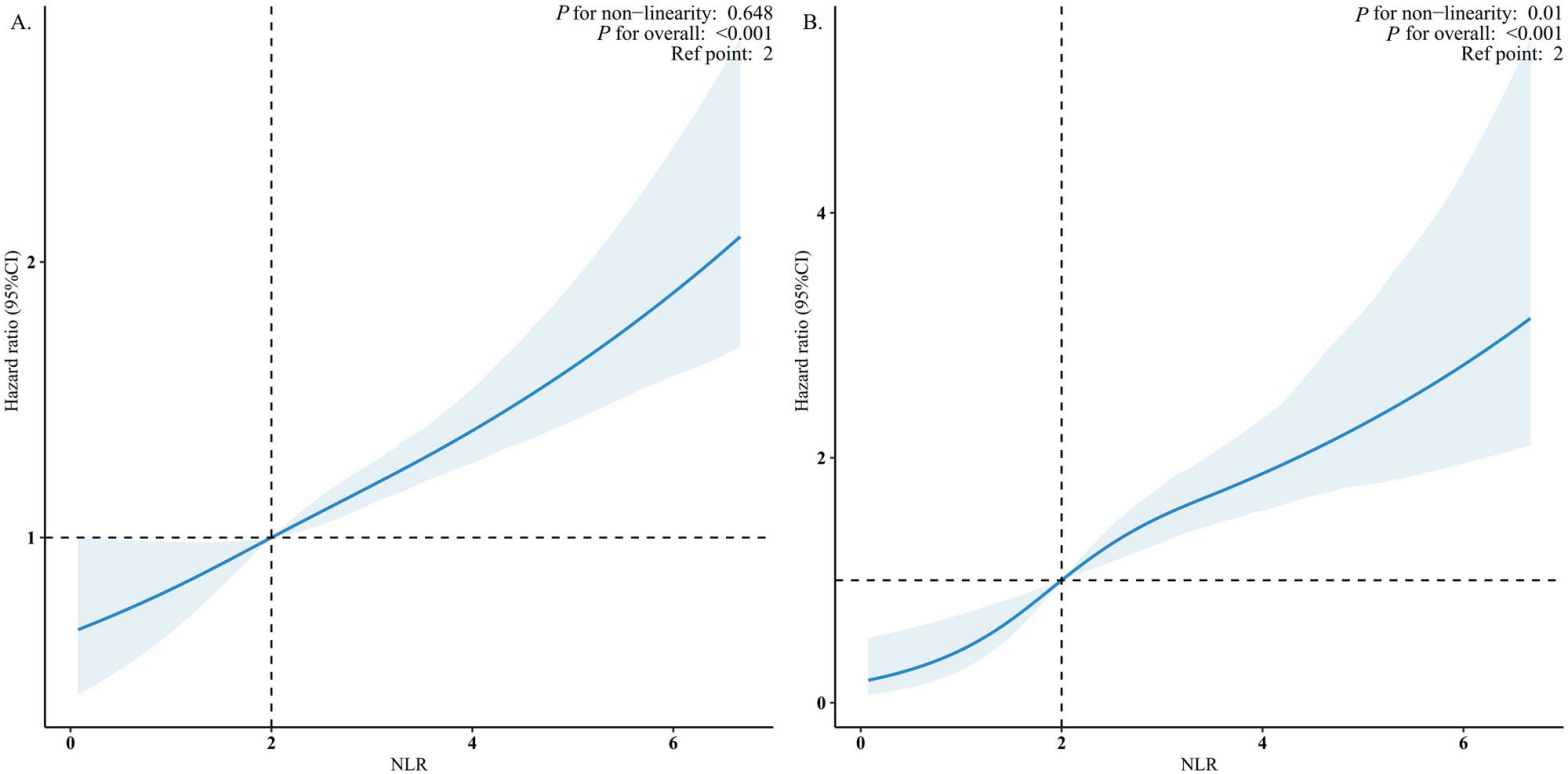
The association of NLR with all‐cause (A) and CVD mortality (B) among depression visualized by restricted cubic spline. HRs were adjusted for age, sex, race, education level, marital status, PIR, BMI, HbA1c, HDL, TC, eGFR, smoking status, alcohol drinking status, diabetes, history of CVD, cancer, antidepressant use, and PHQ_9 score as a continuous variable. Abbreviation: NLR, neutrophil‐to‐lymphocyte ratio; PIR, the ratio of family income to poverty; BMI, body mass index; HDL, high‐density lipoprotein cholesterol; TC, total cholesterol; eGFR, estimated glomerular filtration rate; CVD, cardiovascular disease; PHQ_9, Patient Health Questionnaire‐9.

This study was carried out over a median duration of 84.0 months (IQR, 46.0–127.0 months), during which there were 1023 deaths (11.7%), with 271 of those (3.1%) attributed to CVD. RCS analysis revealed a substantial linear positive correlation between NLR and all‐cause mortality (non‐linear test *p *= 0.648; Figure 2). In the unmodified model (Model 1), a unit increase in NLR correlated with a 32% rise in the risk of mortality from all causes (HR 1.32, 95% CI 1.25–1.38). After accounting for various factors, this correlation remained evident; in Model 4, the likelihood of all‐cause mortality rose by 17% (HR 1.17, 95% CI 1.12–1.23) for each unit increase in NLR (Table 2). Variance inflation factors for all covariates were below 5, indicating no significant multicollinearity in the fully adjusted model (Table S2). The proportional hazards assumption was satisfied, as all Schoenfeld residual tests yielded *p* > 0.05, indicating no significant time‐dependency of the hazard ratios and supporting the validity of the reported estimates (Table S3).

**TABLE 2:** The relationships between NLR and mortality in depression^a^.

	Model 1		Model 2		Model 3		Model 4	
	HR (95% CI)	*p*‐value	HR (95% CI)	*p*‐value	HR (95% CI)	*p*‐value	HR (95% CI)	*p*‐value
**All‐cause mortality**								
NLR	1.32 (1.25, 1.38)	< 0.001	1.17 (1.10, 1.24)	< 0.001	1.17 (1.11, 1.23)	< 0.001	1.17 (1.12, 1.23)	< 0.001
NLR category								
Lower NLR	Reference		Reference		Reference		Reference	
Higher NLR	2.48 (2.05, 3.00)	< 0.001	1.78 (1.49, 2.12)	< 0.001	1.69 (1.43, 2.01)	< 0.001	1.63 (1.36, 1.95)	< 0.001
**CVD mortality**								
NLR	1.39 (1.31, 1.48)	< 0.001	1.21 (1.12, 1.31)	< 0.001	1.25 (1.17, 1.34)	< 0.001	1.27 (1.19, 1.36)	< 0.001
NLR category								
Lower NLR	Reference		Reference		Reference		Reference	
Higher NLR	3.73 (2.79, 4.99)	< 0.001	2.56 (1.87, 3.51)	< 0.001	2.44 (1.79, 3.33)	< 0.001	2.44 (1.77, 3.36)	< 0.001

^a^ Model 1: unadjusted; Model 2: adjusted for age, sex, race, education level, marital status, and PIR; Model 3: adjusted for model 2 plus BMI, HbA1c, HDL, TC, eGFR, smoking status, and alcohol drinking status; Model 4: adjusted for model 3 plus diabetes, history of CVD, cancer, antidepressant use, and PHQ_9 score as a continuous variable.

Abbreviations: 95% CI, 95% confidence interval; BMI, body mass index; CVD, cardiovascular disease; eGFR, estimated glomerular filtration rate; HDL, high‐density lipoprotein cholesterol; HR, hazard ratio; NLR, neutrophil‐to‐lymphocyte ratio; PHQ_9, Patient Health Questionnaire‐9; PIR, the ratio of family income to poverty; TC, total cholesterol.

**FIGURE 3:**
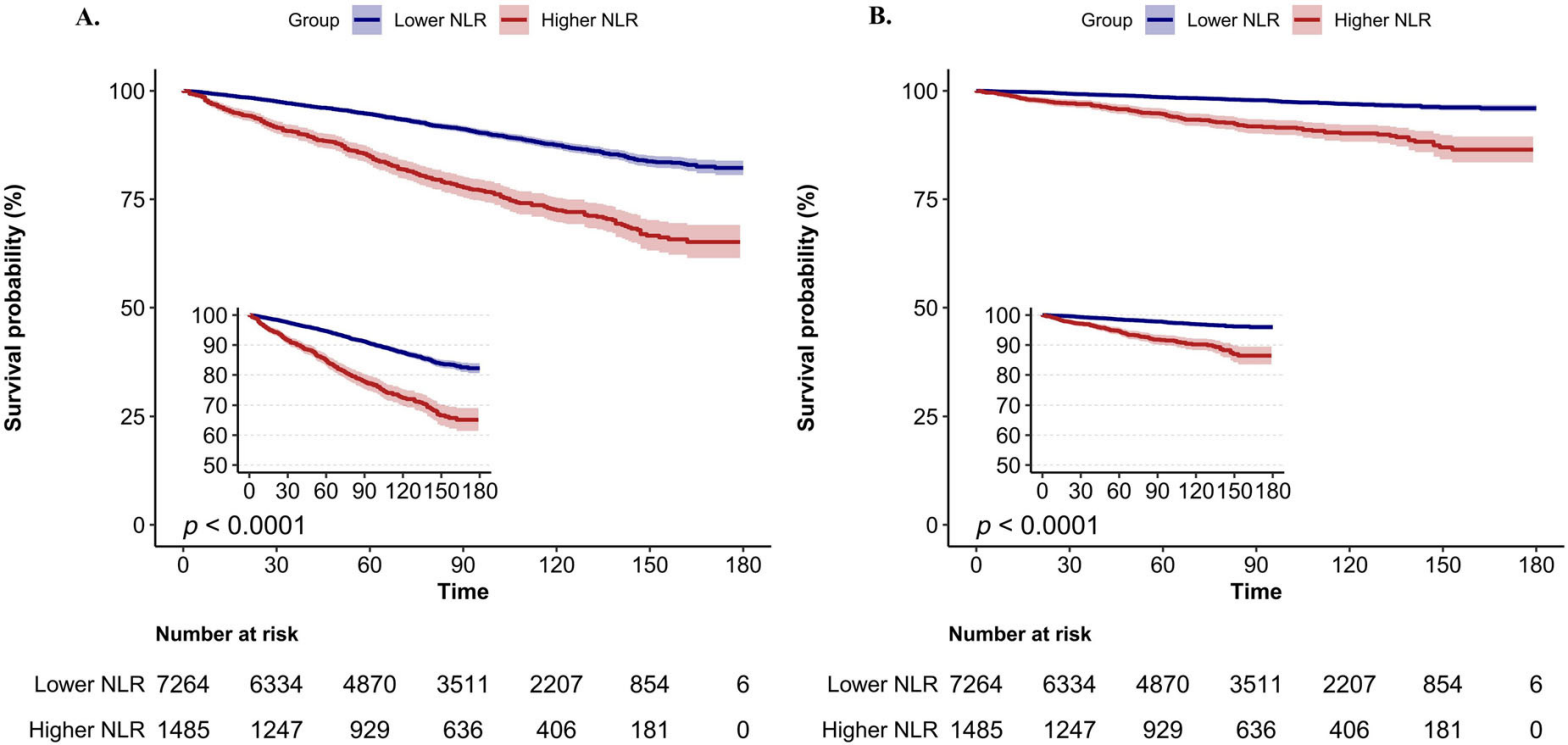
Kaplan–Meier curves of the survival rate with higher (> 3.06) and lower (≤ 3.06) NLR values. (A): All‐cause mortality; (B): CVD mortality.

The analysis of the survival curves indicated that the survival rate was significantly reduced in the group exhibiting a higher NLR when compared to the group with a lower NLR (*p < *0.0001) (Figure 3). When assessing NLR as a categorical variable, it became evident that individuals in high NLR category showed significant correlations in Model 1 (HR 2.48, 95% CI 2.05–3.00), Model 2 (HR 1.78 95% CI 1.49–2.12), Model 3 (HR 1.69, 95% CI 1.43–2.01), and Model 4 (HR 1.63, 95% CI 1.36–1.95). These findings collectively indicate an increased risk of mortality (all *p < *0.05), as illustrated in Table 2. This trend underscores a notable rise in mortality rates from all causes among subjects with elevated NLR values.

**FIGURE 4:**
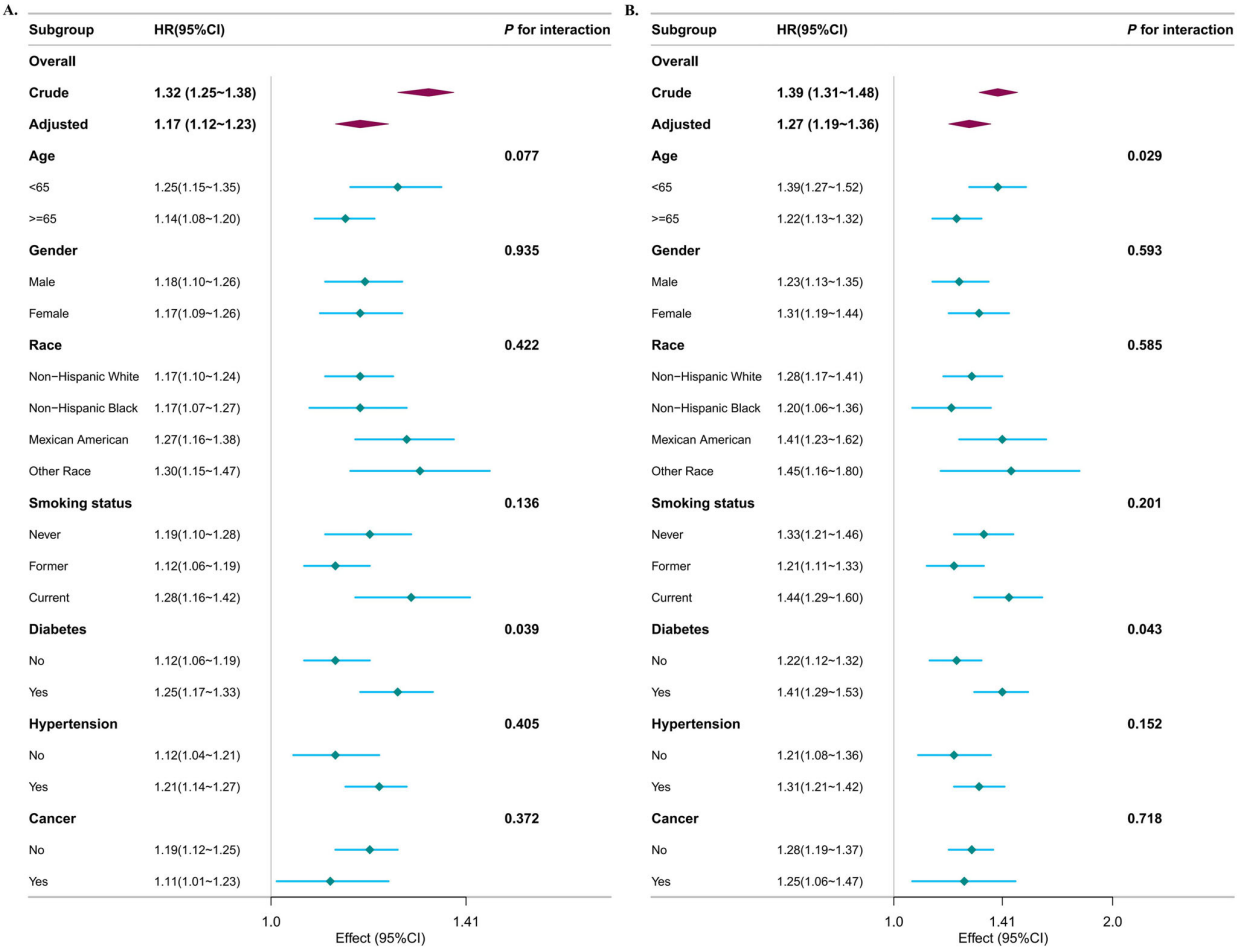
Association between NLR with all‐cause (A) and CVD mortality (B) among those with depression in NHANES 2005–2018. Each stratification was adjusted for age, sex, race, education level, marital status, PIR, BMI, HbA1c, HDL, TC, eGFR, smoking status, alcohol drinking status, diabetes, history of CVD, cancer, antidepressant use, and PHQ_9 score as a continuous variable except the stratification factor itself. The HRs are represented by diamonds, and the horizontal lines denote the 95% CIs. Abbreviation: NLR, neutrophil‐to‐lymphocyte ratio; CVD, cardiovascular disease; PIR, the ratio of family income to poverty; BMI, body mass index; HDL, high‐density lipoprotein cholesterol; TC, total cholesterol; eGFR, estimated glomerular filtration rate; PHQ_9, patient health questionnaire‐9.

Within the subgroup that has diabetes, a more significant rise in mortality rates linked to NLR levels was noted. No substantial interactions between NLR and the other stratified variables were identified (*p* for interaction > 0.05) (Figure 4).

### 3.3 NLR and CVD Mortality

The investigation employing RCS revealed a nonlinear relationship between NLR and the likelihood of mortality attributed to CVD (*p* for non‐linearity = 0.01) (Figure 2). In baseline model (Model 1), elevated NLR levels correlated positively with a heightened risk of mortality associated with CVD (HR 1.39, 95% CI 1.31–1.48) (Table 2). Following comprehensive multivariable adjustments (Model 4), an increase of one unit in NLR led to a 27% rise in the probability of death from CVD (HR: 1.27, 95% CI 1.19–1.36) (Table 2).

Analysis of survival curves demonstrated that participants exhibiting elevated NLR values had a notable reduction in survival rates when compared to those with lower NLR levels (*p < *0.0001) (Figure 3). Additionally, the Cox proportional hazards model supported these results, emphasizing a marked rise in CVD mortality among people exhibiting elevated NLR values. The HRs were recorded as 3.73 (95% CI 2.79–4.99) in Model 1, 2.56 (95% CI 1.87–3.51) in Model 2, 2.44 (95% CI 1.79–3.33) in Model 3, and 2.44 (95% CI 1.77–3.36) in Model 4 (Table 2).

Analyses were performed using stratification, taking into account variables like age, gender, race, smoking habits, diabetes, hypertension, and cancer. Patterns that were consistent were observed throughout all strata. Notably, significant interactions between NLR and the assessed traits were absent, except for individuals aged under 65 and those with diabetes (Figure 4).

### 3.4 Sensitivity Analysis

A sensitivity analysis using quartile‐based categorization revealed a significant dose‐response relationship between NLR and both all‐cause and cardiovascular mortality (*p* for trend < 0.001). In the fully adjusted Model 4, individuals in the highest quartile of NLR (Q4, NLR > 2.7) had significantly increased risks of all‐cause mortality (HR 1.65, 95% CI 1.33–2.07) and cardiovascular mortality (HR 4.11, 95% CI 2.36–7.17) compared to those in the lowest quartile (Q1) (Table S4). The maxstat‐derived cutoff of 3.06 falls within this high‐risk quartile, supporting its biological and statistical relevance.

## 4 Discussion

Our research found a strong correlation between elevated NLR levels in individuals suffering from depressive disorders and the likelihood of mortality due to all causes as well as CVD. The NLR acts as a simple and reliable inflammatory indicator, holding considerable significance in terms of prognostic prediction.

Existing research indicates that inflammatory indicators are significant in evaluating depressed symptoms, assessing treatment effectiveness, and forecasting prognosis (Costello et al. 2019; Harsanyi et al. 2022; Mazza et al. 2018). Nonetheless, systematic assessment of inflammatory cytokines encounters practical constraints, including elevated prices and limited accessibility. Conversely, the identification of peripheral blood leukocytes and their subtypes (neutrophils, lymphocytes, etc.) is more cost‐effective and clinically viable, and these parameters effectively indicate the inflammatory condition typical of several chronic illnesses (Aydin Sunbul et al. 2016).

Neutrophils are pivotal in several pathophysiological processes, serving as essential regulators of both innate and adaptive immunity (Jaillon et al. 2013). As the primary effector cells of the initial immune response, they attract other immune cells to the lesion site via chemokine secretion (Margraf et al. 2022). Reactive oxygen species (ROS) generated by activated neutrophils may induce oxidative stress, resulting in the destruction of biomolecules such as proteins, lipids, and DNA, a process potentially pertinent to the development of depressive disorders (Bhatt et al. 2020; Vorobjeva et al. 2017). Lymphocytes, a main subset of peripheral blood leukocytes, participate in the adaptive immune response and possess immunomodulatory capabilities. NLR consolidates the quantitative data of these two cell types, offering a straightforward and efficient biological metric for evaluating the systemic inflammatory condition of individuals with depression (Sonnenberg and Hepworth 2019).

The significant connection between the elevated NLR and mortality risk in depressed individuals offers further evidence for the “inflammation hypothesis” (Miller et al. 2009). This idea posits that persistent inflammatory conditions may intensify mental symptoms via microglial activation and vascular endothelial impairment (Colasanto et al. 2020). Related findings indicate that treatment approaches targeting inflammatory pathways may enhance prognosis by restoring immunological homeostasis (Bai et al. 2020).

This study identified NLR as a significant predictor of mortality risk, with increased levels correlating with worse outcomes, indicating that routine monitoring of this measure may facilitate the early identification of high‐risk patients and enhance prognostic evaluation. These findings possess significant therapeutic translational significance and provide a theoretical foundation for the formulation of targeted anti‐inflammatory intervention strategies for individuals with depression.

Our research's primary strengths include (1) a diverse and nationally representative sample; (2) comprehensive longitudinal follow‐up data; (3) the application of multiple statistical methods to address confounding variables; (4) the first systematic examination of the link between NLR as well as long‐term mortality risk in people with depression; and (5) the utilization of sampling weights to enhance the external validity of the findings.

The present study has several limitations. Firstly, the NHANES database lacks critical clinical information, such as systemic inflammatory indicators and infection status, which may affect the interpretation of the findings. Secondly, only NLR was assessed, while other inflammation‐related metrics, including monocyte/lymphocyte ratio, platelet‐related parameters, and inflammatory markers such as IL‐6 as well as CRP, were omitted. Additionally, the diagnosis of depression was based on the PHQ‐9 scale (sensitivity 0.88, specificity 0.80) rather than the DSM‐5 criteria (Muñoz‐Navarro et al. 2017). Future research should include more extensive inflammatory indicators and standardized diagnostic techniques to clarify the intricate link between depression and inflammation.

Another potential limitation is that we did not exclude participants with early deaths or extreme NLR values. As such, individuals at very high short‐term risk, possibly due to acute illness or severe systemic inflammation, may have contributed disproportionately to the observed associations, potentially inflating the hazard ratios. While our findings remain robust after extensive multivariable adjustment, the generalizability of the results to long‐term mortality risk should be interpreted with caution. Future studies may consider sensitivity analyses that exclude individuals with early deaths or extreme baseline NLR values to further validate these associations over extended follow‐up periods.

## 5 Conclusion

Our study discovered that an increased NLR in individuals with depressive disorders was strongly correlated with the probability of all‐cause as well as CVD mortality. The NLR serves as a straightforward and dependable inflammatory measure with significant prognostic predictive value. Incorporating this statistic into clinical evaluation systems may enhance risk classification and guide precision medicine choices. This biomarker may identify high‐risk groups that may benefit from focused therapies, thereby enhancing the efficiency of healthcare resource allocation. Subsequent research should investigate the association between NLR and other inflammatory markers, as well as determine the best threshold criteria for depressed individuals to enhance the current risk assessment framework.

## Author Contributions

**Yue Chai**: conceptualization, methodology, formal analysis, writing–original draft, writing–review and editing, project administration. **Guoxin Wang**: investigation, data curation, writing–original draft, writing–review and editing, supervision. **Shumin Zhu**: conceptualization, formal analysis, investigation, visualization. **Congzhen Wei**: resources, validation. **Runsen Du**: data curation. **Zining Liu**: conceptualization. **Shuo Zhao**: conceptualization. **Li Yang**: conceptualization, supervision, funding acquisition. **Yulan Geng**: conceptualization, supervision, project administration.

## Conflicts of Interest

The authors declare no conflicts of interest.

## Peer Review

The peer review history for this article is available at https://publons.com/publon/10.1002/brb3.70983.

## Supporting information




**Supplementary Material**: brb370983‐sup‐0001‐SuppMat.doc

## Data Availability

The data used in this study are publicly available from the National Health and Nutrition Examination Survey (NHANES), conducted by the Centers for Disease Control and Prevention (CDC). Researchers and the public can freely access and download the datasets from the official NHANES website: https://www.cdc.gov/nchs/nhanes/
