# Supplementary material for: Association of Neutrophil‐to‐Lymphocyte Ratio With All‐Cause and Cardiovascular Mortality Among Individuals With Depression: A Large‐Scale Cohort Study
Source: Brain Behav. 2025 Oct 15;15(10):e70983. doi: 10.1002/brb3.70983 (PMC12528552; doi:10.1002/brb3.70983)
Supplement: Supplementary file 1 — Supplementary Material: brb370983‐sup‐0001‐SuppMat.doc [file BRB3-15-e70983-s001.doc]

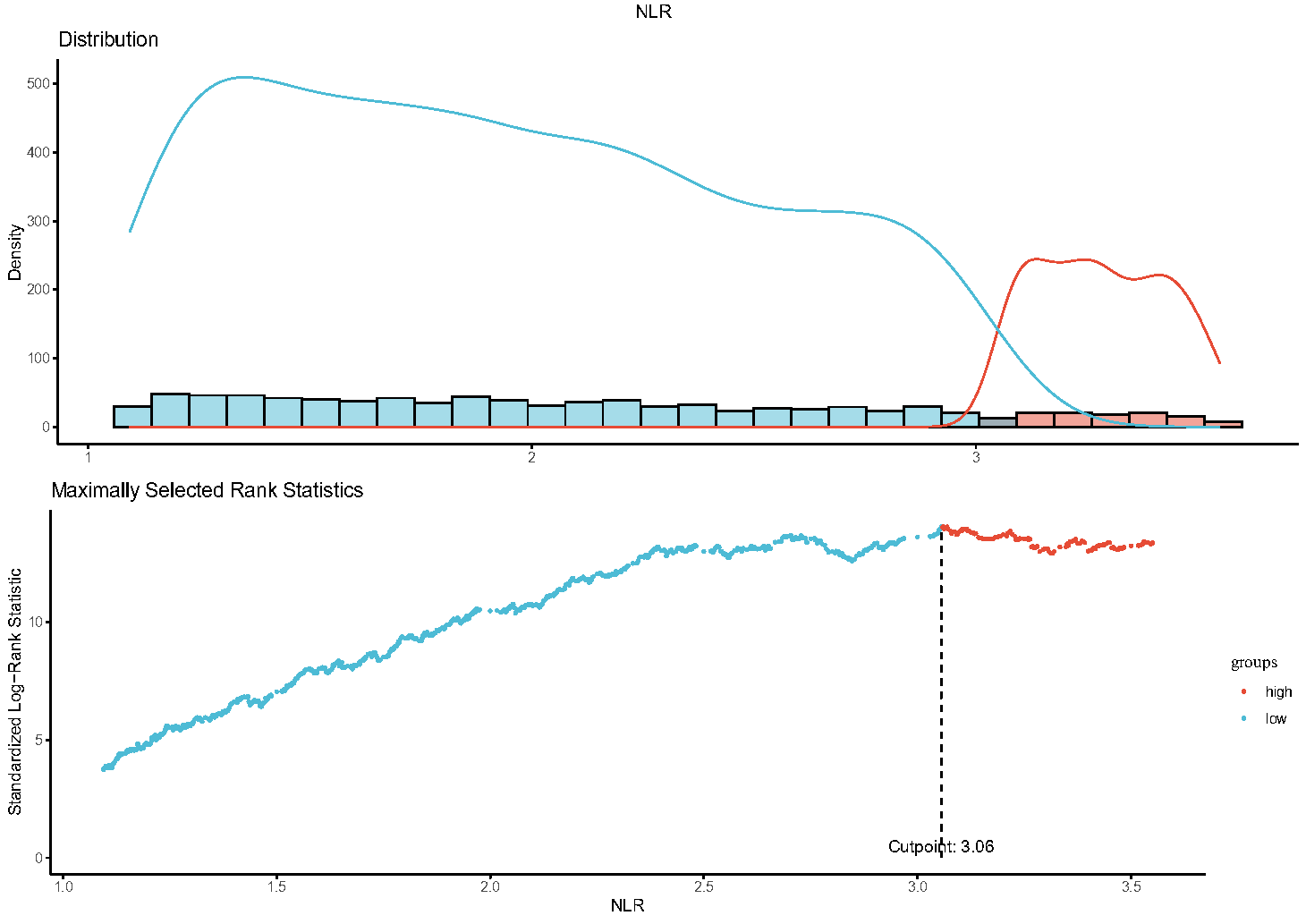


**Figure S1.** The cutoff threshold was derived using the maximum chosen rank statistics from the 'maxstat' program.

**Abbreviation:** NLR, neutrophil-to-lymphocyte ratio.

**Table S1.** Generic names of antidepressant drugs used by participantsin the NHANES 2005-2018.

| Amitriptyline | Imiprammne |
| --- | --- |
| Bupropion | Mirtazapine |
| Buspirone | Nefazodone |
| Citalopram | Nortriptyline |
| Escitalopram | Paroxetine |
| sertraline | Protriptyline |
| Fluoxetine | Venlafaxine |

**Table S2.** The variance inflation factors (VIF) of covariates.

| Variables | VIF |
| --- | --- |
| Age, years | 1.86 |
| Gender, % | 1.33 |
| Race/Ethnicity, % | 1.58 |
| Education level, % | 1.37 |
| Marital status, % | 1.11 |
| PIR, % | 1.24 |
| BMI, kg/m^2^ | 1.28 |
| HbA1c, % | 1.51 |
| HDL, mmol/L | 1.37 |
| TC, mmol/L | 1.18 |
| eGFR, mL/min/1.73m^2^ | 1.62 |
| Smoking status, % | 1.46 |
| Drinking status, % | 1.13 |
| Diabetes, % | 1.65 |
| History of CVD, % | 1.21 |
| Cancer, % | 1.10 |
| Antidepressant use, % | 1.06 |
| PHQ_9 | 1.08 |

Abbreviation: PIR, the ratio of family income to poverty; BMI, body mass index; HDL, high-density lipoprotein cholesterol; TC, total cholesterol; eGFR, estimated glomerular filtration rate; CVD, cardiovascular disease; PHQ_9, Patient Health Questionnaire-9;

**Table S3.** Proportional Hazards Assumption Testing Using Schoenfeld Residuals for All-Cause and CVD Mortality.

| **Variables** | **P value** | |
| --- | --- | --- |
|  | **All-Cause Mortality** | **CVD Mortality** |
| Age, years | 0.142 | 0.199 |
| Gender, % | 0.102 | 0.613 |
| Race/Ethnicity, % | 0.568 | 0.694 |
| Education level, % | 0.169 | 0.737 |
| Marital status, % | 0.983 | 0.253 |
| PIR, % | 0.636 | 0.558 |
| BMI, kg/m^2^ | 0.346 | 0.094 |
| HbA1c, % | 0.685 | 0.759 |
| HDL, mmol/L | 0.312 | 0.966 |
| TC, mmol/L | 0.127 | 0.513 |
| eGFR, mL/min/1.73m^2^ | 0.439 | 0.669 |
| Smoking status, % | 0.847 | 0.088 |
| Drinking status, % | 0.162 | 0.228 |
| Diabetes, % | 0.490 | 0.147 |
| History of CVD, % | 0.506 | 0.225 |
| Cancer, % | 0.833 | 0.742 |
| Antidepressant use, % | 0.319 | 0.105 |
| PHQ_9 | 0.817 | 0.168 |
| NLR | 0.063 | 0.983 |

Abbreviation: PIR, the ratio of family income to poverty; BMI, body mass index; HDL, high-density lipoprotein cholesterol; TC, total cholesterol; eGFR, estimated glomerular filtration rate; CVD, cardiovascular disease; PHQ_9, Patient Health Questionnaire-9; NLR, neutrophil-to-lymphocyte ratio.

**Table S4.** The relationships between NLR and mortality in depression.^a^

|  | **Model 1** |  | **Model 2** |  | **Model 3** |  | **Model 4** |  |  |
| --- | --- | --- | --- | --- | --- | --- | --- | --- | --- |
|  | **HR**  **(95% CI)** | **P value** | **HR**  **(95% CI)** | **P value** | **HR**  **(95% CI)** | **P value** | **HR**  **(95% CI)** | **P value** | |
| **All-cause mortality** |  |  |  |  |  |  |  |  | |
| NLR | 1.32  (1.25, 1.38) | <0.001 | 1.17  (1.10, 1.24) | <0.001 | 1.17  (1.12, 1.23) | <0.001 | 1.17  (1.12, 1.23) | <0.001 | |
| NLR category |  |  |  |  |  |  |  |  | |
| Q1 | Reference |  | Reference |  | Reference |  | Reference |  | |
| Q2 | 1.00  (0.77, 1.31) | 0.98 | 1.04  (0.80, 1.34) | 0.791 | 0.97  (0.76, 1.23) | 0.807 | 0.95  (0.75, 1.21) | 0.683 | |
| Q3 | 2.48  (2.05, 3.00) | 0.029 | 1.09  (0.82, 1.45) | 0.55 | 1.01  (0.77, 1.33) | 0.932 | 1.00  (0.76, 1.31) | 0.999 | |
| Q4 | 2.48  (2.05, 3.00) | <0.001 | 1.90  (1.49, 2.42) | <0.001 | 1.72  (1.38, 2.16) | <0.001 | 1.65  (1.33, 2.07) | <0.001 | |
| P for trend | <0.001 |  | <0.001 |  | <0.001 |  | <0.001 |  | |
| **CVD mortality** |  |  |  |  |  |  |  |  | |
| NLR | 1.39  (1.31, 1.48) | <0.001 | 1.21  (1.12, 1.31) | <0.001 | 1.25  (1.17, 1.34) | <0.001 | 1.27  (1.19, 1.36) | <0.001 | |
| NLR category |  |  |  |  |  |  |  |  | |
| Q1 | Reference |  | Reference |  | Reference |  | Reference |  | |
| Q2 | 1.66  (0.85, 3.22) | 0.135 | 1.83  (0.96, 3.51) | 0.067 | 1.64  (0.87, 3.09) | 0.124 | 1.61  (0.86, 3.02) | 0.136 | |
| Q3 | 2.49  (1.40, 4.43) | 0.002 | 2.07  (1.13, 3.77) | 0.018 | 1.74  (0.93, 3.24) | 0.083 | 1.78  (0.95, 3.34) | 0.074 | |
| Q4 | 6.97  (4.03, 12.06) | <0.001 | 4.9  (2.84, 8.45) | <0.001 | 4.09  (2.37, 7.06) | <0.001 | 4.11  (2.36, 7.17) | <0.001 | |
| P for trend | <0.001 |  | <0.001 |  | <0.001 |  | <0.001 |  | |
| **^a^** Model 1: unadjusted.  Model 2: adjusted for age, sex, race, education level, marital status, and PIR.  Model 3: adjusted for model 2 plus BMI, HbA1c, HDL, TC, eGFR, smoking status, alcohol drinking status.  Model 4: adjusted for model 3 plus diabetes, history of CVD, cancer, antidepressant use, and PHQ_9 score as a continuous variable.  NLR was divided into four levels by quartile (Q1 ≤ 1.5; 1.5 < Q2 ≤ 2.0; 2.0 < Q3 ≤ 2.7; Q4 > 2.7).  **Abbreviations:** HR, hazard ratio; 95% CI, 95% confidence interval; NLR, neutrophil-to-lymphocyte ratio; CVD, cardiovascular disease; PIR, the ratio of family income to poverty; BMI, body mass index; HDL, high-density lipoprotein cholesterol; TC, total cholesterol; eGFR, estimated glomerular filtration rate; PHQ_9, Patient Health Questionnaire-9 | | | | | | | | |  |
